# Training a Nonclinician to Become a Leader in Transdisciplinary Clinical Research: Clinical Observerships

**DOI:** 10.23937/2469-5831/1510005

**Published:** 2015-09-28

**Authors:** Douglas D Gunzler, James C Spilsbury, Michael Fu, Susannah Rose, Neal V Dawson, Shirley Moore, Thomas E Love

**Affiliations:** 1 Center for Health Care Research and Policy, Case Western Reserve University, USA; 2 Department of Medicine, Case Western Reserve University, USA; 3 Center for Clinical Investigation, Case Western Reserve University, USA; 4 Department of Electrical Engineering and Computer Science, Case Western Reserve University, USA; 5 Physical Medicine & Rehabilitation, Case Western Reserve University, USA; 6 Department of Veterans Affairs Medical Center, Cleveland FES Center of Excellence, Louis Stokes Cleveland, USA; 7 Department of Bioethics, Cleveland Clinic, USA; 8 Department of Medicine, Cleveland Clinic Lerner College of Medicine, Case Western Reserve University, USA; 9 Department of Epidemiology & Biostatistics, Case Western Reserve University, USA; 10 Frances Payne Bolton School of Nursing, Case Western Reserve University, USA

## Abstract

As institutions and funders expand their efforts toward multidisciplinary, interdisciplinary, and even transdisciplinary research, there is a substantial need for scientists who can lead such efforts. The traditional image of a leader of transdisciplinary clinical research is a quantitatively savvy clinician conducting research at some point along the continuum from the “bench to the bedside.” However, this is not the only alternative. Nonclinician scientists can also provide effective leadership in multidisciplinary clinical research teams. To foster the development of such nonclinician-leaders, we suggest the completion of a comprehensive clinical observership as an appropriate and valuable training activity. The clinical setting can foster unique insight, providing invaluable training for leadership in translational research. Observing both providers and patients immerses nonclinician scientists in the health care experience, providing them with direct exposure to clinicians, patients, and patients’ families, and thereby motivating patient-centered research.

## Introduction

Research institutions and funders have increased interest in cross-disciplinary research [1,2], which enables researchers to branch out, gain new perspectives, and pursue novel ideas [3]. This can in turn shed light on human and organizational processes that research isolated to a single field might otherwise overlook.

Commonly, three types of approaches - multidisciplinary, interdisciplinary and transdisciplinary - are used as models for involving multiple disciplines in research. In a multidisciplinary approach, researchers draw appropriately from multiple disciplines to redefine problems and reach solutions but remain within the boundaries of the different disciplines. An interdisciplinary approach integrates separate disciplines into a coordinated and coherent whole. A transdisciplinary approach transcends the pre-conceived boundaries of the different disciplines and refers to learning that is authentic and relevant to the real world [3]. Rather than being confined by traditional ways of thinking about problems, learning in the transdisciplinary approach is supported and enriched by them.

The traditional image of a transdisciplinary clinical researcher is a quantitatively savvy clinician engaging in research “from the bench to the bedside.” However, a nonclinician, both with and without direct experience with patients relevant to the current research questions, may also play an important role as a leader in translational clinical research. Of course, nonclinicians do occasionally lead healthcare research projects. For example, nonclinicians have developed new analytic methods with clear clinical application or as a result of a clinical application on a R01 study [4–6].

Becoming a leader in transdisciplinary clinical research involves understanding, welcoming and enhancing the contributions of different disciplines. For starters, clinical research projects that involve any aspect of the collection, aggregation or interpretation of quantitative clinical information require statistical analyses. Such analyses are most appropriately and commonly conducted in collaboration with a biostatistician or other statistical scientist [7]. Similarly, studies may involve a component for gathering qualitative information, in which collaboration between a clinician and qualitative research specialist would likely be appropriate.

Since a nonclinician often lacks formal training in or deep knowledge of the clinical field under study, the clinician commonly provides input for the context of the study and implications of the quantitative or qualitative information. In such a study, a nonclinician may learn important details through discussion with the clinician and other health-care providers, reading pertinent books or articles, and online research.

In principle, the clinician’s input takes shape, beyond scholastic learning, through personal interactions with patients with the condition(s) of interest [8]. Clinicians may have the opportunity to grasp how patients experience the condition literally in all senses (sight, sound, smells, taste and touch). Deep personal experience often provides strong motivation for the clinician to study the condition in the first place [9]. Similarly, having a personal understanding of patient behavior will influence how the clinician designs and conducts a typical patient visit to obtain useful information (i.e. measures, diagnoses and scales) about symptoms and treatment effectiveness.

What about researchers who do not provide clinical services to persons, or who may be clinicians but unfamiliar with the clinical objective of the research? How might they gain access to this “experiential knowledge” that arises from clinical interaction with patients and their families? We propose an underutilized educational activity, the clinical observership that will allow a nonclinician to experience these personal interactions in an approach that transcends the boundaries of typical methodological work. The clinical observership provides four important results for the nonclinician towards becoming a leader across multiple disciplines: 1) exposure to the personal, motivating stories of individual patients, 2) introduction of methodological skillsets into the clinical setting, 3) creation of a reservoir of experience, which can be drawn upon again in the future to guide cross-disciplinary work with other clinicians or mentees, 4) exposure to professionals of multiple disciplines, which can be used for identifying members for a transdisciplinary research team.

Previously, Walter et al. and Greenwald et al. described accounts of nonclinicians taking part in clinical rounds [10,11]. We extend this previous work by proposing 1) the clinical observership as an activity to train a nonclinician for a transdisciplinary leadership role, 2) a more focused observership for education for a particular research study, enabling a nonclinician to attain a more thorough understanding of the condition of interest as revealed through the experiences of doctors and patients, 3) the use of the observership for shadowing other aspects of care (i.e. clinician meetings, group therapy). Here, we discuss how this educational activity can be achieved using examples of nonclinical scientists’ clinical observerships: a statistician’s observership of patients with multiple sclerosis (MS) for a study examining depression issues in MS patients; and an electrical engineer’s observership of stroke survivors for developing computer-enhanced approaches for restoring motor function after stroke. We also discuss how this educational activity can be beneficial for a clinician in the example of a clinical social worker/bioethicist’s observership of heart failure and of Palliative Medicine clinicians. In each case, the observership helped provide greater understanding between disciplines, thus providing a more transdisciplinary approach to conducting research.

## Designing a Clinical Observership of Patients for a Nonclinican

### Multiple sclerosis-depression study for a biostatistician

Our first example arises from a study using structural equation modeling methodology to disentangle the overlapping symptoms (i.e. fatigue) of MS and depression [12,13]. This work is funded by Cleveland’s Clinical and Translational Science Collaborative (CTSC) KL2 training program (http://casemed.case.edu/ctsc/education/kl2/), designed for training clinical researchers to become leaders in multidisciplinary research. The statistical scientist conducting the study needed a better understanding of the relationship between MS and depressive symptoms for a transdisciplinary approach to the research. A carefully designed clinical observership was tailored to meet these specific educational needs.

Setting up such a clinical observership required a supervising specialist in the condition under study. In the MS-depression study, we sought both a MS specialist and psychiatrist to supervise different aspects of the observership. The supervisor allowed the observer to shadow them in meetings with other clinicians and staff and as they treated patients. Further, the supervisor participated in reflective discussions with the observer about individual patients through the day or at the end of the observership sessions.

The supervisor also scheduled a full rotation through other departments in addition to the department in the discipline of medicine relevant for investigating the study’s research questions. For example, in the MS-depression study, the clinical professions of psychology and occupational rehabilitation therapy were included.

Each institution had necessary protocol (forms, agreements, etc.) to permit the nonclinician to observe patients in the clinical setting. In addition, patients must formally consent to an observer’s presence during their interactions with health care providers.

The clinical observership for the MS-depression study was 12 days in length. Seven days (one day a week for seven consecutive weeks) were spent at the Cleveland Clinic’s Mellen Center for Multiple Sclerosis, under the supervision of a Board Certified Staff Neurologist with expertise in MS. Along with observing consultation appointments with the neurologist and patients, this observership also included a rotation through other clinical disciplines at the Mellen Center, specifically psychology and occupational therapy.

Five days of the clinical observership took place at the MetroHealth Medical Center, Cleveland, Ohio, the main campus of a county-wide, public healthcare system. At this location, under the supervision of a psychiatrist, the statistician rounded with the inpatient consultation liaison psychiatry team. This rotation involved patients with depression in addition to a variety of other medical/psychiatric illnesses outside of the realm of multiple sclerosis. Of special interest to the statistician for later use in modeling, this observership component provided greater understanding of how fatigue manifests differently in a patient diagnosed with both depression and MS versus depression alone.

The statistician’s observership led to specific material output, including journal notes describing anecdotes about patients, different clinicians’ reflections about depression in MS patients, and the statistician’s own growing insights for the MS-depression study. With a particular study in mind, some discussions with the clinicians focused specifically on the overlapping symptoms of MS and depression and the trajectory of depression in MS patients. These discussions informed the later development of a transdisciplinary conceptual model of the overlap of depression and MS, as well as recommendations for implementing the analytic results in clinical application [12]. Finally, the information and particular anecdotes were incorporated into seminars regarding the study at national meetings for biostatisticians and other clinical researchers.

### Stroke rehabilitation study for an electrical engineer

A second example of a nonclinican scientist benefitting from a clinical observership involved an electrical engineer who was also a CTSC KL2 scholar. He was working to translate his expertise in human-computer interaction to develop computer-enhanced approaches for restoring motor function in patients post-stroke. His training to gain clinical exposure to individual stroke survivors and the course of their care consisted of observing the outpatient and inpatient stroke services at the Cleveland Clinic and MetroHealth Medical Center for 60 hours over the course of three months. The observership was designed to cover the entire spectrum of stroke care. This included a neurointerventional radiology service at stroke onset, the physical medicine and rehabilitation inpatient stroke floor where acute care and therapy occurs, and the outpatient clinic where subacute, chronic, follow-up, and rehabilitation care are performed. These rotations were designed to provide exposure to the wide array of clinicians in stroke care teams. The stroke care teams consisted of attending physicians, resident physicians, rehabilitation psychologists, social workers, nurses, and therapists (recreation, music, occupational, physical, and speech pathology).

Additionally, he was exposed to the application of traditionally nonclinical approaches to stroke motor relearning while assisting his KL2 mentors (a biomedical engineer and a physician with expertise in critical care, pulmonary and internal medicine) in their ongoing clinical trials. These trials involved using neuromuscular electrical stimulation to treat hemiplegia after stroke.

The experiences from these rotations helped him gain insight into the challenges in stroke rehabilitation faced by both clinicians and patients. Also, the experiences helped him identify a clinically-relevant problem to fuel his research effort. He is currently developing and conducting clinical trials of a home-based intervention to treat hemiplegia using video games and neuromuscular electrical stimulation.

### Palliative medicine for patients with heart failure

While we have discussed the experience of nonclinicians above, it is important to note that clinicians can also benefit from such an observership. For instance, this can occur when clinicians would like to learn more about a specific patient population or an unfamiliar care environment in order to improve their research. Our third example is a health services clinician-researcher, also a CTSC KL2 scholar, who is a clinical social worker and bioethicist. She specifically wanted to expand her knowledge of Palliative Medicine and heart failure for a new research project she was conducting in patients with advanced heart failure that integrates Palliative Medicine into their care. Although this researcher has extensive clinical experience providing psychotherapy to patients with cancer and their families, and in conducting clinical bioethics consultations, she wanted to expand her knowledge for the research project in order to better understand the unique needs of heart failure patients and their families. Based on the information collected through direct experience with patients and providers, she sought to develop a more transdisciplinary conceptual model of how Palliative Medicine could be adapted from its more common application within cancer to heart failure patients.

She identified three mentors, two in heart failure and one in Palliative Medicine. Through their guidance, she attended heart failure rounds, and also Palliative Medicine rounds, in which detailed clinical cases were discussed by physicians, nurses, social workers, surgeons and other specialists. Furthermore, given her unique role as a clinician in bioethics, she was able to contribute ideas to the care team of heart failure and Palliative Medicine clinicians, including physicians and nurses. She used these collaborative care interactions to gain valuable knowledge about patient, family and clinicians’ perspectives and roles, and with this input was able to develop the needed conceptual framework or model to guide her research study.

## Discussion

As illustrated by the examples above, the clinical observership of patients holds four important advantages for research. First, direct exposure to patients provides personal anecdotes that can be used as motivation for research [8]. These personal anecdotes can also be recalled when shaping the study setting and refining analyses. In the MS-depression study the observership led to a better understanding of the relationship between MS and depressive symptoms for a transdisciplinary approach to the statistical modeling. In the stroke recovery research, the observership proved fruitful for developing the aims of a follow-up study. In the Palliative Medicine example, the observership allowed a researcher to expand her clinical expertise to a new field for interdisciplinary research.

Second, by establishing a relationship with clinicians in a particular clinical setting (i.e. MS Center, Inpatient Stroke Services), new, different scenarios to implement ideas for improving care come about that are in the methodological skillset of the nonclinician (i.e. structural equation modeling, mediation analysis, longitudinal data analysis, electrical engineering), but typically outside of the clinician’s realm of knowledge [12,13]. For instance, the MS specialists at the Mellen Center were introduced to the benefits of using SEM methodology with patient reported outcomes recorded in their electronic health records database. The measures could be adjusted to understand the specific latent dimension of interest (i.e. the PHQ-9 could be adjusted to isolate the depression latent dimension in the PHQ-9, for a more pure estimate of the MS patient’s mood) using SEM methodology [12]. They could envision using such an adjusted measure along with their expert judgment to further aid patients. Thus, this second advantage is of mutual benefit for both the nonclinician and the clinician (and ultimately, the patient).

Third, this observership adds a new, transdisciplinary experience to the list of experiences as a nonclinician [3]. This is an experience that can be drawn on in the future when determining how to design a study or what assumptions to make for statistical modeling. The picture the nonclinician can build upon for the context of applied analyses in the future involves a real clinical setting with patient experience and care in mind. Nonclinicians will find themselves asking better research questions, given their more in depth knowledge about the clinician-patient experience. For example, in the MS-depression study, the observership leads to a more in depth understanding of how fatigue manifests in a MS patient. Thus, there is more knowledge to draw on to develop a tool for understanding the overlap of fatigue in MS-depression patients. Further, in relating to clinicians, the nonclinician now has shared in a clinical experience. The nonclinician can continue to draw on this experience in educating and mentoring clinicians or other nonclinicians.

Fourth, the clinical observership provides working knowledge for team science building. After the clinical observership, the nonclinician can better identify team members for interdisciplinary research. For example, the MS-depression clinical observership involved physicians, psychiatrists, occupational therapists, and psychologists, and thus exposure to their individual expertise. Similarly, the clinical observership can serve as an initial basis for outlining the role of representations of the essential disciplines for a new research study. In the Palliative Medicine example, the clinical observership mentors became key research collaborators.

When developing observerships, there are advantages of working in larger institutions, such as the institutions participating in the CTSC KL2 training program, where the potential opportunities for clinical observerships may be greater. However, smaller institutions may still afford opportunities; e.g. collaboration with local health clinics/departments, observing local health services, participating in clinical rounds, and participating in other institutions’ grand rounds or relevant meetings via remote internet technologies (or travelling directly to other institutions).

Determining to what degree an individual clinical observership achieves the four advantages outlined above is a measure of effectiveness. The experience will most likely lead to a short term emotional surge due to the inspiring stories of individual patients. Thus, when evaluating the experience, it is important for the nonclinician to take a step back and note if goals were objectively achieved. One way to do this is to record notes about the observership in a journal and then reflect on these notes a month or so removed from the experience. As previously discussed, since the observership leads to some material output (journal notes, papers and seminars), at least some of the benefits of the observership can be objectively assessed. Also, the supervisors/mentors of the observership could themselves participate in a formal evaluation of the observership. This might involve their objective assessment if the formal goals of the observership were met as well as recommendations of future activities to further enhance the education of the nonclinician/mentee. Since many of the benefits of the clinical observership have future implications, rather than only present implications, it may not be entirely feasible to explicitly evaluate the entire experience in the short term.

In short, the clinical observership immerses a nonclinician directly in the clinical setting in a role that crosses disciplinary boundaries (i.e. biostatistician, electrical engineer, and clinician). This new role provides invaluable experience towards becoming a leader in transdisciplinary research.
